# Adaptive space search-based molecular evolution optimization algorithm

**DOI:** 10.1093/bioinformatics/btae446

**Published:** 2024-07-23

**Authors:** Fei Wang, Xianglong Cheng, Xin Xia, Chunhou Zheng, Yansen Su

**Affiliations:** Key Laboratory of Intelligent Computing and Signal Processing of Ministry of Education, Information Materials and Intelligent Sensing Laboratory of Anhui Province, School of Artificial Intelligence, Anhui University, Hefei 230601, China; Institute of Artificial Intelligence, Hefei Comprehensive National Science Center, Hefei 230088, China; Key Laboratory of Intelligent Computing and Signal Processing of Ministry of Education, Information Materials and Intelligent Sensing Laboratory of Anhui Province, School of Artificial Intelligence, Anhui University, Hefei 230601, China; Institute of Artificial Intelligence, Hefei Comprehensive National Science Center, Hefei 230088, China; Key Laboratory of Intelligent Computing and Signal Processing of Ministry of Education, Information Materials and Intelligent Sensing Laboratory of Anhui Province, School of Artificial Intelligence, Anhui University, Hefei 230601, China; Institute of Artificial Intelligence, Hefei Comprehensive National Science Center, Hefei 230088, China; Key Laboratory of Intelligent Computing and Signal Processing of Ministry of Education, Information Materials and Intelligent Sensing Laboratory of Anhui Province, School of Artificial Intelligence, Anhui University, Hefei 230601, China; Institute of Artificial Intelligence, Hefei Comprehensive National Science Center, Hefei 230088, China; Key Laboratory of Intelligent Computing and Signal Processing of Ministry of Education, Information Materials and Intelligent Sensing Laboratory of Anhui Province, School of Artificial Intelligence, Anhui University, Hefei 230601, China; Institute of Artificial Intelligence, Hefei Comprehensive National Science Center, Hefei 230088, China

## Abstract

**Motivation:**

In the drug development process, a significant portion of the budget and research time are dedicated to the lead compound optimization procedure to identify potential drugs. This procedure focuses on enhancing the pharmacological and bioactive properties of compounds by optimizing their local substructures. However, due to the vast and discrete chemical structure space and the unpredictable element combinations within this space, the optimization process is inherently complex. Various structure enumeration-based combinatorial optimization methods have shown certain advantages. However, they still have limitations. Those methods fail to consider the differences between molecules and struggle to explore the unknown outer search space.

**Results:**

In this study, we propose an adaptive space search-based molecular evolution optimization algorithm (ASSMOEA). It consists of three key modules: construction of molecule-specific search space, molecular evolutionary optimization, and adaptive expansion of molecule-specific search space. Specifically, we design a fragment similarity tree in a molecule-specific search space and apply a dynamic mutation strategy in this space to guide molecular optimization. Then, we utilize an encoder–encoder structure to adaptively expand the space. Those three modules are circled iteratively to optimize molecules. Our experiments demonstrate that ASSMOEA outperforms existing methods in terms of molecular optimization. It not only enhances the efficiency of the molecular optimization process but also exhibits a robust ability to search for correct solutions.

**Availability and implementation:**

The code is freely available on the web at https://github.com/bbbbb-b/MEOAFST.

## 1 Introduction

In drug development, it is crucial to optimize molecules to enhance their pharmacological and bioactive properties, and weaken their unsatisfactory properties, such as toxicity and adverse reactions (Bulbule *et al.* 2023, Mohammed *et al.* 2023). The change of a molecule’s property is essentially bound up with the modification of its chemical structures. Molecule optimization is the key step to improve the properties of a molecule while maintaining structural similarity to the original molecule (Hoffman *et al.* 2021). Traditional experiment-driven methods are time-consuming and energy-intensive (Bohacek *et al.* 1996), and the huge chemical search space (about $10^{60}$ molecules; Polishchuk *et al.* 2013) further increases the difficulty of searching for ideal molecules. Therefore, it is important to develop efficient lead molecule optimization strategies to accelerate the discovery of novel molecules for medicinal purposes.

Recent advancements in machine learning have led to the development of deep learning-based models and combinatorial optimization models. Deep learning-based models optimize molecules with unsatisfactory properties by projecting these molecules to a latent space and then search for molecules with satisfactory properties in the latent space (Fu *et al.* 2021). The combinatorial optimization models guided by deep neural networks are posed based on historical chemistry data to help medicinal chemists to efficiently design molecules. These models generate potential candidates matching molecular property distributions of the molecules with satisfactory properties in databases (Gómez-Bombarelli *et al.* 2018). Recent work has exploited different molecule generation techniques for multi-properties optimization, such as a retrieval-based framework (RetMol; Wang *et al.* 2023) and a tree generative model (MSTG; Wang *et al.* 2022). Those methods require smooth and differentiated latent spaces for molecules with unsatisfactory properties and those with satisfactory properties, but the limited availability of training data (especially molecules with satisfactory multi-properties) hinders their training and application for molecule optimization (Fu *et al.* 2022).

Various combinatorial optimization-based methods have shown notable advantages in molecule optimization tasks, as they are not reliant on large-scale task-related data and can handle the indeterminacy of structure combinations in discrete chemical space (Kirkpatrick and Ellis 2004). These models are predominantly utilized in fragment-based drug design process, which has a much smaller search space compared to atom-based drug design ($10^{7}$ versus $10^{60}$) (Grantham *et al.* 2022). Generally, combinatorial optimization-based molecule optimization methods optimize substructures of molecules in a combinatorial and stochastic way and do not require a large molecular database relevant to a given optimization task (Jensen 2019, Jules *et al.* 2020, Spiegel and Durrant 2020). For instance, MolFinder is a genetic algorithm-based global optimization algorithm, where a global search on discrete chemical space is performed by the crossover and mutation operations on the SMILES representation of molecules, and multiple properties are combined by using weighted summation to update the population (Kwon and Lee 2021). Further, a graph-based molecular Pareto optimization model is proposed for molecular multi-objective optimization, where new molecules are generated from randomly sampled populations (Verhellen 2022). Most of these models exhibit random-walk behavior to a certain extent and apply trial-and-error strategies to search for valid molecules in the discrete chemical space, which may affect the efficiency of molecular optimization.

Recently, deep neural networks have been utilized to direct the combinatorial optimization algorithms, addressing the issue of random-walk behavior problem (You *et al.* 2018, Zhou *et al.* 2019, Grantham *et al.* 2022). For instance, a general graph convolutional network-based model (GCPN; You *et al.* 2018) and a molecule deep q-networks (MolDQN; Zhou *et al.* 2019) make use of deep neural networks to direct the search directions. Additionally, methods such as a Markov molecular sampling method (MARS; Xie *et al.* 2021), a multi-constraint molecule sampling approach (MIMOSA; Fu *et al.* 2021), and a search-driven approach based on Monte Carlo tree search (MolSearch; Sun *et al.* 2022) apply Markov chain Monte Carlo method and graph neural networks to effectively explore the distribution of targets. Furthermore, a deep generative model and multi-objective evolutionary computation are integrated for the deep evolutionary learning of molecule optimization (Grantham *et al.* 2022).

The mentioned molecule optimization algorithms have demonstrated competitiveness for molecules whose properties are easy to be optimized. The performance of most existing algorithms, however, will considerably deteriorate as the number of properties increases and the difficulty of optimization increases. First, the sub-structures used for optimizing a molecule are often limited to a predefined and fixed fragment library, which leads to a limited exploration of chemical space. In GB-GA, the crossover and mutation operations were performed by choosing sub-structures of the first 1000 molecules by the penalized logP score in the database of commercially available compounds for virtual screening (ZINC; Irwin and Shoichet 2005) to alter a graph representation of molecules.

In MARS, the top 1000 frequently occurring fragments with no more than 10 heavy atoms in a manually curated database of bioactive molecules with drug-like properties (ChEMBL; Gaulton *et al.* 2017) were extracted to construct the fragment vocabulary. Most of these fragment/sub-structure libraries are extracted from a limited set of “excellent” molecules (i.e. molecules with satisfactory properties) or include limited frequently occurring fragments. As a result, they may lack certain desired substructures for certain properties (You *et al.* 2018).

Previous efforts have focused on expanding those libraries. For instance, a low-frequency masking-based algorithm (LFM; Podda *et al.* 2020) and a deep evolutionary learning algorithm (DEL; Grantham *et al.* 2022) have been posed on plenty of fragments broken from 227 945 to 383 790 molecules in the ZINC dataset and the PubChem bioassay dataset (PCBA), respectively. Besides, MolSearch uses more than 600 000 independent fragments and more than 1 000 000 transformation rules to approximate the whole search space (Sun *et al.* 2022). However, it is still difficult to receive new useful fragments for improving the properties of molecules. A large number of useless fragments during the optimization reduce the efficiency of methods.

To address those challenges, we propose an adaptive space search-based molecular evolution optimization algorithm, termed ASSMEOA. ASSMEOA constructs a constantly updated molecule-specific fragment library for each molecule, which enables the incorporation of new useful fragments to facilitate the generation of molecules with satisfactory properties and is the key to the proposed model. Specifically, the main contributions of the proposed ASSMEOA are summarized as follows.

- We propose a strategy to construct a molecule-specific fragment search space to address the limited and inefficient exploration of chemical space. Each molecule-specific fragment library is initially included the decomposition fragments of molecules with satisfactory properties in the database, and then is enlarged by adding the fragments from the new generated molecules with satisfactory properties in each iteration.
- We propose a molecule optimization algorithm to optimize molecules efficiently. We also propose a dynamic mutation strategy by replacing the fragments of a molecule with those in the molecule-specific fragment search space.
- Our ASSMEOA achieves the best optimization results compared to six benchmark algorithms in three multi-objective optimization tasks. It is able to optimize the lead molecule properties.

## 2 ASSMEOA model

**Figure 1.**
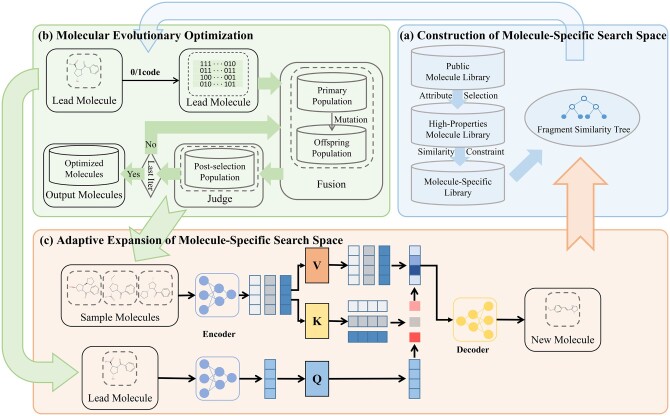
The architecture of ASSMEOA. Module (a): Construction of molecule-specific fragment search space; Module (b): Molecule evolutionary optimization; Module (c): Adaptive expansion of molecule-specific search space.

In this section, we illustrate the details of the proposed ASSMOEA. As shown in Fig. 1, ASSMOEA mainly consists of three modules: (a) the construction of a molecule-specific fragment search space (i.e. a fragment library related to a given molecule), (b) the molecule evolutionary optimization, and (c) the adaptive expansion of molecule-specific fragment search space.

Specifically, the construction of molecule-specific fragment library involves creating distinct search spaces of fragments for each molecule to optimize. Based on the similarity between fragments within the search space, the connections between fragments are established using a tree structure called the fragment similarity tree. The molecule evolutionary optimization is based on the embeddings in Module (a) to optimize the molecule toward better properties progressively. A dynamic mutation strategy is proposed to guide the molecule’s mutation operation during the evolution process. Module (c) involves a cross-attention mechanism to generate novel molecules and dynamically expand the molecule-specific fragment search space. Those three modules are processed iteratively to optimize molecules.

### 2.1 Construction of molecule-specific fragment search space

In this subsection, we construct a molecule-specific fragment search space, as illustrated in Module (a), Fig. 1. The search space is a library of fragments. To generate the fragment library, an intermediate molecule library *S* is first identified from the ZINC database. According to the objectives of a specific task, molecules with properties that exceed a certain threshold are collected to form *S*.

For a given molecule *x*, its molecular fingerprint similarities with other molecules in *S* are calculated. We select the top *N* molecules with the highest similarities and decompose them into fragments. In the decomposing procedure, we apply Kawai’s method (Kawai *et al.* 2014) to generate fragments from a molecule. The fragments are further labeled and assembled based on the treatment of fragments in Niclas Ståhl’s work (Ståhl *et al.* 2019). Specifically, given a molecule, all single bonds extending from each ring atom in the molecule are cut off and the locations of breakpoints are marked as an attachment point for subsequent fragment assembly. Assuming that the molecule splits into *r* fragments with a total of *t* attachment points, the *r* fragments can be replaced by any number of fragments if the total number of attachment points is constant.

**Figure 2.**
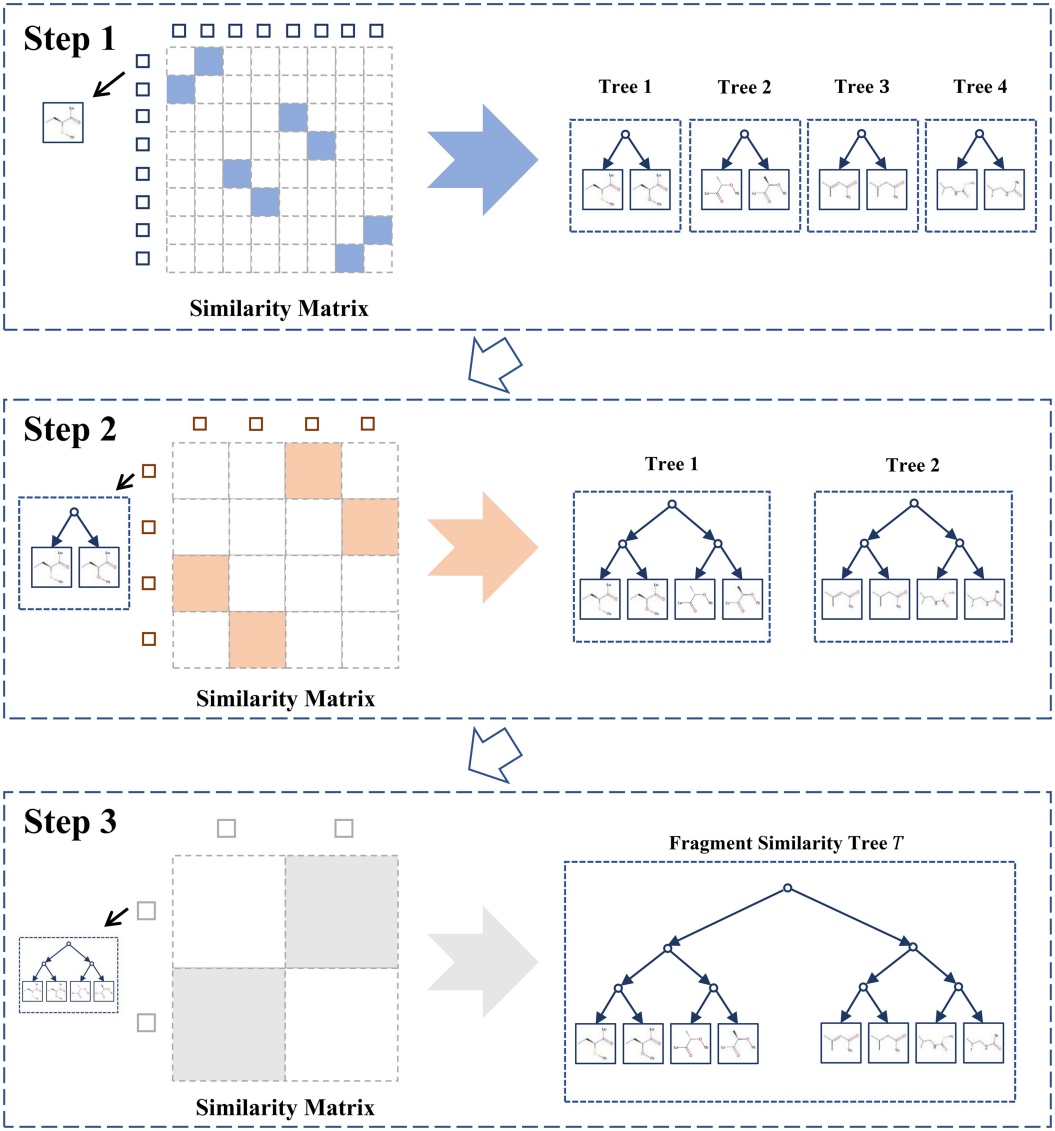
The fragment similarity tree is constructed with a 2D similarity matrix on the left side of each step to record the similarity between fragments and fragments, and a stepwise composition of the fragment similarity tree on the right side.

The fragments of *N* molecules are collected to form an initial search space $S_{x}$ of molecule *x*. They reflect the similarity between fragments in $S_{x}$, we construct a fragment similarity tree *T* for further analysis. A tree example of eight fragments is illustrated in Fig. 2.

In Step 1, we construct a similarity matrix of fragments and generate a forest of binary trees. According to the maximum common substructure Tanimoto similarity TanimotoMCS (TS) (Boström *et al.* 2006) and Levenshtein distance methods (Levenshtein *et al.* 1966), the similarity of two fragments are calculated as follows:

(1) $$A_{\mathrm{sim}}(i,j)=\max(\text{Tan}(f_{i},f_{j}),\mathrm{Lev}(f_{i},f_{j}))$$

where $A_{\mathrm{sim}}$ is the similarity matrix, $f_{i}$ and $f_{j}$ are the *i*th and *j*th fragments, 1≤i≤n, 1≤j≤n, i≠j, *n* is the number of fragments, *Tan* and *Lev* represent Tanimoto similarity and Levenshtein distance, respectively.

Based on the similarity matrix, those *n* fragments are divided into ⌈n/2⌉ groups. The maximum size of a group is 2, that two most similar fragments are placed into the same group. Each group has a corresponding tree. Two fragments are identified as the left and right leaf nodes, and a virtual parent node is added. If *n* is odd, a tree contains only one fragment at its left node. In Step 1, a forest of ⌈n/2⌉ trees is constructed.

In Step 2, we combine the trees in the forest. A similarity matrix between two trees $T_{i}$ and $T_{j}$ is defined as follows:

(2) $$A_{\mathrm{sim}}^{\left(T_{i},T_{j}\right)}(i,j)=\max(\text{Tan}(f_{i},f_{j}),\mathrm{Lev}(f_{i},f_{j}))$$

where $f_{i}$ is the *i*th fragment in $T_{i}$, and $f_{j}$ is the *j*th fragment in $T_{j}$.

The similarity between the two trees $T_{i}$ and $T_{j}$ is defined as the maximum similarity between the fragments in each tree:

(3) $$\mathrm{sim}(T_{i},T_{j})=\max(A_{\mathrm{sim}}^{\left(T_{i},T_{j}\right)})$$

Two most similar trees are placed into a group to form a new tree, that a novel virtual parent node is added. Then, the number of trees is reduced from ⌈n/2⌉ to ⌈n/4⌉. The Step 2 is repeated iteratively until two trees are remained.

**Figure 3.**
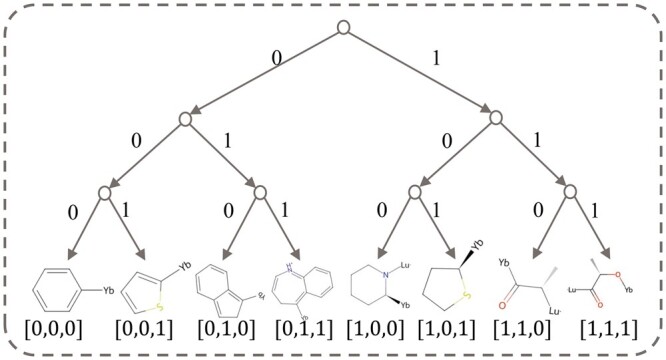
A fragment similarity tree of depth 4 consisting of eight fragments.

In Step 3, we generate a final similarity tree *T* of all fragments. The two remained trees in Step 2 are combined to form *T*. Since it is a binary tree, its depth is $\left\lceil {\;\text{log}\;}_{2}n\right\rceil$. In the fragment similarity tree *T*, the left path has a code “0” and the right path has a code “1.” Starting from the root node, every leaf node has a corresponding path, consisting of “0” and “1.” The path sequence is defined as the embedding of the fragment whose dimension is $\left\lceil {\;\text{log}\;}_{2}n\right\rceil$. An instance is illustrated in Fig. 3, in which the first fragment from the left is encoded as [0, 0, 0] and the first fragment from the right is encoded as [1, 1, 1].

### 2.2 Molecular evolutionary optimization

After finishing the construction of molecule-specific search space for each lead molecule, we optimize the molecules using an evolutionary algorithm, as described in Module (b), Fig. 1. It aims to find molecules with improved properties while maintaining similarity to the lead molecule.

In this module, the lead molecule is decomposed into a number of fragments. Each fragment has a unique embedding with the length of $\left\lceil {\;\text{log}\;}_{2}n\right\rceil$, based on the fragment similarity tree in Module (a). The longer the common prefix they have, the more similar the fragments are. The embedding of a molecule consists of the embeddings of the fragments it has, and its length is $m\times \left\lceil {\;\text{log}\;}_{2}n\right\rceil$, where *m* is the number of fragments.

The initial population is constructed based on the lead molecule. We randomly change the binary values on the lead molecule embedding to generate 2*M* different embeddings. We apply the Pareto optimal algorithm and population crowding ordering to select *M* embeddings. The initial population consists of *M* molecule embeddings.

Two mutation operators are applied to the initial population to generate offspring solutions. Since a molecule consists of *m* fragment, the mutation operators act on all fragment embeddings of a molecule. One operator changes the binary value on each position of an embedding with the same probability. Since the embedding of a fragment is based on its position in the fragment similarity tree, the influence of a mutation on a rear position is much smaller than that on a front position.

Another operator changes the binary value on each position of a fragment embedding with different probabilities. Those probabilities are calculated as p+qi, where *i* represents the *i*th position in the fragment embedding, *p* and *q* are two hyper-parameters. It tends to mutate on rear positions, compared with the first operator.

To adjust the offspring embedding solutions during the iteration, we assign two weights $1-\text{cos}(\frac{\pi }{2}*\frac{\mathrm{Iter}}{\mathrm{Ite}r_{\max}})$ and $\text{cos}(\frac{\pi }{2}*\frac{\mathrm{Iter}}{\mathrm{Ite}r_{\max}})$ to change the rate of two operators, where *Iter* and $\mathrm{Ite}r_{\max}$ represent the current number of iterations and the maximum number of iterations, respectively. As the iteration continues, Module (b) tends to balance the mutation probability between front positions and rear positions.

After mutation operators in each iteration, *M* novel molecules are constructed based on those *M* novel embeddings. The molecules with satisfied properties are collected to form a group of sample molecules in Module (c). For a given task and property, the threshold is defined as $\delta_{p}-\mathrm{Iter}*\eta_{p}$, where $\delta_{p}$ and $\eta_{p}$ are hyper-parameters of property *p*. Specifically, the value of $\mathrm{Ite}r_{\max}$ can increase to ensure the minimum number of sample molecules is *k*. At the end of iteration, the final *M* molecules are outputted as optimized molecules.

### 2.3 Adaptive expansion of molecule-specific search space

In this subsection, we use an encoder–fusion–decoder structure to generate novel molecules, as illustrated in Module (c), Fig. 1.

In Module (c), two kinds of molecules are fed into two encoders, including a lead molecule and *k* sample molecules. The sample molecules are generated in Module (b), as described in Section 2.2. The encoder structure is designed in the ChemFormer model, which is trained on the billion-level ZINC dataset and achieves state-of-the-art generative performance.

The two encoders output three types of feature vectors, including query vector, key vector, and value vector, as shown in Fig. 1. Then, we apply a pre-trained information fusion structure from RetMol (Wang *et al.* 2023) to concatenate those feature vectors together.

The integrated vector is fed into a decoder structure, which is also designed in ChemFormer. Both the dimension of the output layer of the decoder and that of the encoder are the same. We use the cross-entropy loss function to train this module.

After the decoder procedure, we generate an embedding of a novel molecule. It is decomposed into fragments and added to the search space in Module (a). The adaptive expansion of molecule-specific search space is achieved.

In the proposed method, the above three modules are processed iteratively. The Module (b) generates fragment embedding in Module (a) and identifies novel molecules for Module (c) to expand the molecule-specific search space in Module (a).

## 3 Experiment and discussion

### 3.1 Method comparison

We compare our ASSMEOA method with six state-of-the-art molecular optimization baseline methods.

*QMO:* It is a query-based molecular generative model (Hoffman *et al.* 2021).

*RetMol:* It is a search-based molecular generative model (Wang *et al.* 2023). It searches a group of molecule samples from the Retrieval Database to direct the pre-trained model to generate a satisfied novel molecule.

*GB-GA:* It is a molecular graph-based genetic algorithm model that constructs the search space based on the substructures and atoms from the first 1000 molecules in the ZINC dataset (Jensen 2019).

*MARS:* It is a Markovian sampling-based model that generates candidate molecules by modifying molecular fragments, where the top 1000 frequently occurring fragments with no more than 10 heavy atoms from the ChEMBL database are extracted to construct the fragment library (Xie *et al.* 2021).

*MolSearch:* It is a search framework for multi-objective molecule generation and optimization using Monte Carlo search trees. It constitutes the search space based on more than 1 million transformation rules and 600 000 pairs of unique fragment pairs extracted from ChEMBL (Sun *et al.* 2022).

*JANUS:* It is a fragment-based multi-population genetic algorithm for the purpose of molecular multi-feature optimization (Nigam *et al.* 2022). It pre-sets an original group of 10 000 molecules, decomposes fragments for mutation operator, and generates novel molecules.

### 3.2 Experimental setup and datasets

We consider the following indicators for evaluation.

- **Similarity (SIM):** The Tanimoto similarity between the lead molecule and an optimized molecule based on molecular fingerprints.
- **Success Rate (SR):** The percentage of lead molecules that can be optimized to molecules with satisfied properties.

In our work, we focus on the molecular properties as follows:

- **Quantitative Estimate of Drug likeness (QED):** It is a quantitative indicator of drug-likeness (Bickerton *et al.* 2012).
- **Dopamine Receptor (DRD2):** An indicator of a molecule’s biological activity against a biological target dopamine type 2 receptor (Comings *et al.* 1996).
- **Penalized LogP (PLogP) and the improvement of PLogP (** ${\text{PLogP}}_{\text{imp}}$ **):** The log of the solute partition ratio between octanol and water minus the synthetic accessibility fraction and the number of long periods (Ertl and Schuffenhauer 2009).
- **Synthetic Accessibility (SA):** It is a score to measure the attribute of synthetic (Ertl and Schuffenhauer 2009).
- **Inhibition of Glycogen Synthase Kinase-3** β **(Inhi-GSK3** β **):** The inhibition score of glycogen synthase kinase-3β (Li *et al.* 2018).

QED, DRD2, PLogP, and SA are non-bioactive objectives, while Inhi-GSK3β is a bioactive objective. The QED, DRD2, and PLogP values of a chemically valid molecule are evaluated by the RDkit toolkit (Landrum 2014). The SA and Inhi-GSK3β are calculated by the TDC toolkit (Huang *et al.* 2021). The QED, DRD2, SA, and Inhi-GSK3β values of a molecule always fall within the range of [0, 1], while the PLogP value does not have an upper bound. Therefore, the improvement of PLogP (namely PLogP_imp) is used as an indicator to measure the optimization performance of molecules.

### 3.3 Multi-property molecule optimization tasks

We design three optimization tasks to evaluate the performance of methods. Two tasks represent non-bioactive optimization and one task represents bioactive optimization. The objective of non-bioactive optimization is several non-bioactive metrics, including QED, PLogP, DRD2, and SIM. The objective of bioactive optimization is bioactive score Inhi-GSK3β, as well as drug-like and synthetically accessible, which are measured by QED and SA, respectively.

#### 3.3.1 Task I: QED+PLogP_imp_+SIM

A molecule is successfully optimized if its output molecule meets the conditions: QED ≥ 0.85, ${\text{PLogP}}_{\text{imp}}\;\geq \;3$, and SIM ≥0.3. In this work, the unoptimized molecules are QED ≤0.8 and PLogP ≤−2 in the ZINC database.

#### 3.3.2 Task II: QED+DRD2+SIM

A molecule is successfully optimized if its output molecule meets the conditions: QED ≥ 0.8, DRD2 ≥ 0.4, and SIM ≥ 0.3. In this work, the unoptimized molecules are QED ≤ 0.8 and DRD2 ≤ 0.02 in the ZINC database.

#### 3.3.3 Task III: QED+Inhi-GSK3β+SA+SIM

A molecule is successfully optimized if its output molecule meets the conditions: QED ≥ 0.7, Inhi-GSK3β ≥ 0.4, SA ≥ 0.7, and SIM ≥ 0.2. In this work, the unoptimized molecules are QED ≤ 0.55, GSK3β ≤ 0.1, and SA ≤ 0.55 in the ZINC database.

In all three tasks, the majority objective is to generate novel molecules with larger attributes and similar to the lead molecule. The larger the value is, the better molecule it is.

### 3.4 Comparison experiments

In the experiments, all datasets are downloaded from the ZINC database, version 15. It consists of 250 000 molecules. Based on the objectives in each task, we construct a molecule library and a corresponding fragment library from the ZINC database. Each library contains the molecules with larger properties. In detail, according to Task I, we select the molecules whose QED ≥ 0.85 and PLogP ≥ 0, then decompose them into fragments and construct a fragment library I. The number of molecules is about 20 000, while that of fragments is 5136. In Task II, we select the molecules whose DRD2 ≥ 0.03 to construct a fragment library II. The number of molecules is about 13 000, while that of fragments is 4884. In Task III, we select the molecules whose QED ≥ 0.75, GSK3β ≥ 0.1, and SA ≥ 0.75 to construct a fragment library III. The number of molecules is about 5000, while that of fragments is 2123.

**Figure 4.**
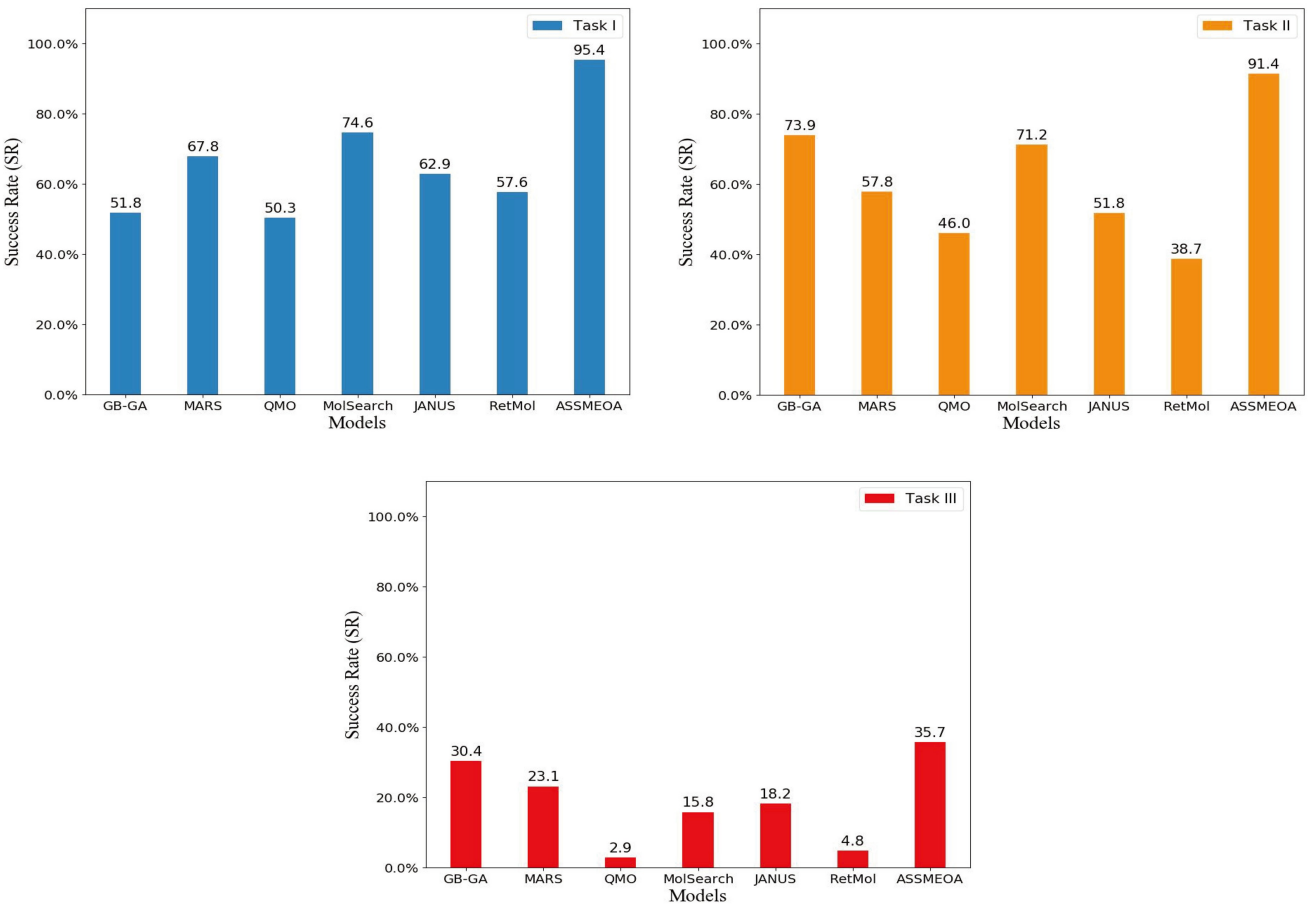
The SR values of all compared models in three tasks.

The first comparison metrics is SR. It indicates the utilization potentiality of a method. We illustrate all SR values of optimizing lead molecules in Fig. 4. Our proposed ASSMEOA method achieves the highest SR in all three tasks. Especially, the SR value is >90% in Task I and Task II. ASSMEOA is better than the second-best method by 20.81% and 17.5% in Task I and Task II, respectively. In Task III, although it is more complex, the lead of ASSMEOA is 35%.

**Figure 5.**
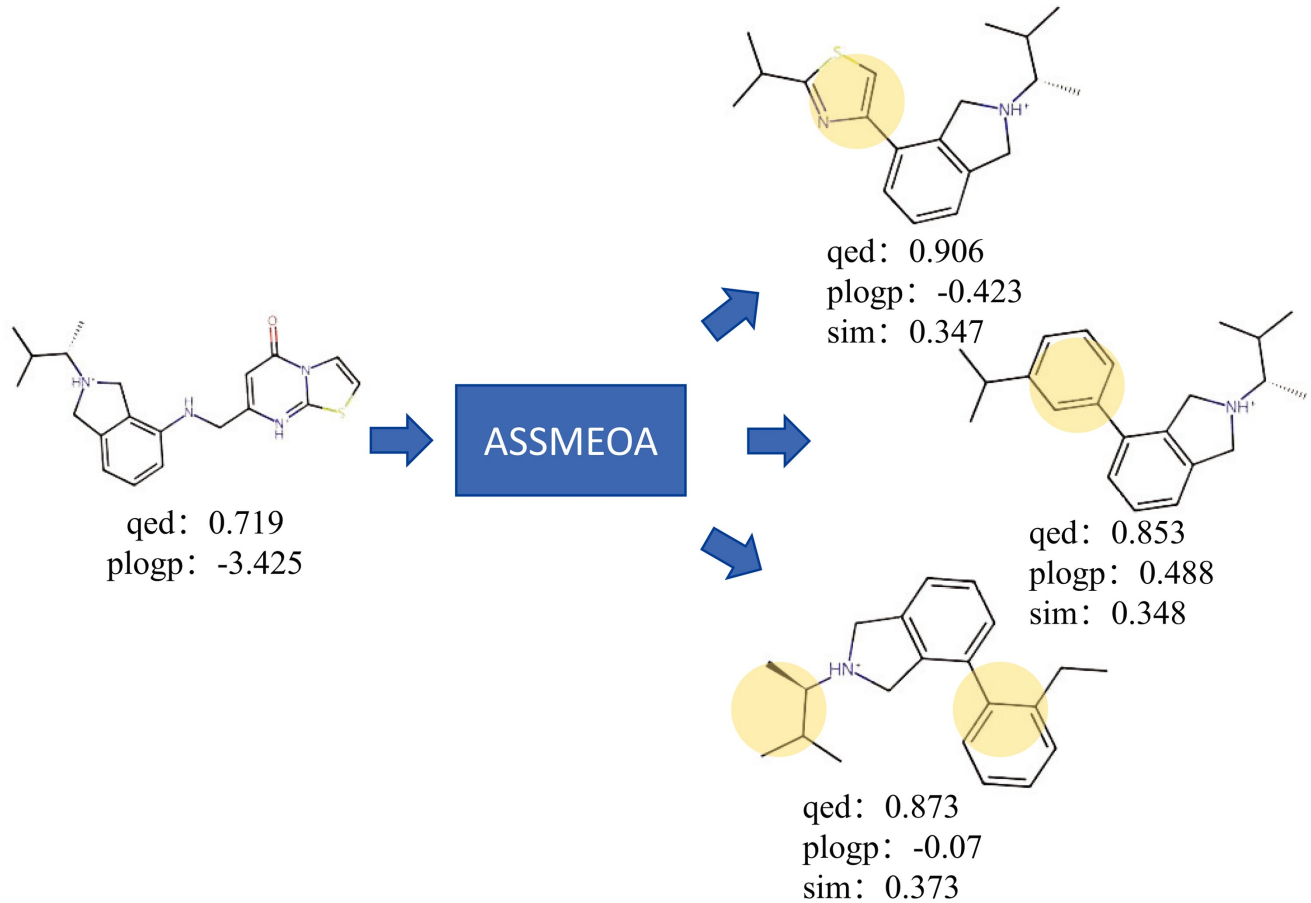
An optimized example with one lead molecule and three optimized results.

Furthermore, an optimization example is shown in Fig. 5. The lead SMILES of the given molecule is CC(C)[C@H](C)[NH+]1Cc2cccc(NCc3 cc(=O)n4ccsc4[nH+]3)c2C1. Only our ASSMEOA can optimize it successfully. One reason is that ASSMEOA generates a number of novel fragments which benefit the optimization process.

The comparison indicates that ASSMEOA has the largest chance to generate an optimized result than all other methods. Moreover, the SRs of QMO and RetMol methods are <5% in Task III. Those two methods can hardly optimize a specific molecule. In the following part, we focus on the comparison of the methods with higher SRs.

The second comparison metrics are molecule properties. To reduce bias, we repeat each method five times and calculate the average value and standard deviation in each task. The results are illustrated in Table 1. In all three tasks, our ASSMEOA achieves the largest values in all properties but SIM.

**Table 1.** Comparison results of ASSMEOA and the compared methods on three tasks.

(a) Task I (QED+PLogP_imp+SIM)			
Model	QED	${\text{PLogP}}_{\text{imp}}$	SIM
GB-GA	0.866 ± 0.082	4.263 ± 1.132	0.364 ± 0.074
MARS	0.892 ± 0.028	4.402 ± 0.940	0.367 ± 0.074
MolSearch	0.892 ± 0.034	3.543 ± 0.676	**0.455 **±** **0.104
JANUS	0.837 ± 0.169	4.178 ± 0.923	0.356 ± 0.095
ASSMEOA	**0.895 **±** **0.029	**4.571 **±** **1.033	0.399 ± 0.081

(b) Task II (QED+DRD2+SIM)			
Model	QED	DRD2	SIM
GB-GA	0.832 ± 0.107	0.607 ± 0.284	0.376 ± 0.119
MARS	0.858 ± 0.049	0.704 ± 0.145	0.328 ± 0.068
MolSearch	0.868 ± 0.042	0.597 ± 0.268	**0.439 **±** **0.127
JANUS	0.831 ± 0.116	0.522 ± 0.214	0.317 ± 0.105
ASSMEOA	**0.871 **±** **0.042	**0.762 **±** **0.185	0.392 ± 0.076

(c) Task III (QED+Inhi-GSK3β+SA+SIM)				
Model	QED	Inhi-GSK3β	SA	SIM
GB-GA	0.724 ± 0.154	0.274 ± 0.174	0.750 ± 0.081	**0.333 **±** **0.168
MARS	0.741 ± 0.082	0.403 ± 0.158	0.732 ± 0.096	0.223 ± 0.065
MolSearch	0.625 ± 0.148	0.218 ± 0.112	0.588 ± 0.072	0.241 ± 0.122
JANUS	0.658 ± 0.248	0.250 ± 0.186	0.612 ± 0.094	0.215 ± 0.064
ASSMEOA	**0.764 **±** **0.131	**0.455 **±** **0.178	**0.760 **±** **0.093	0.250 ± 0.122

The best values are in bold.

In the compared methods, MolSearch is the closest method to our ASSMEOA. One possible reason is that MolSearch constructs a search space based on the substructures and transformation rules in the ChEMBL database. It gives MolSearch more possibility to optimize molecules with different substructures. However, it cannot explore the whole search space in a single iteration, which limits its performance.

The performances of MARS and GB-GA are unstable in Tasks I and II, while MARS shows better performance than GB-GA in Task I, and GB-GA is better in Task II. One possible reason for the observed results is that MARS and GB-GA inherently select fragments from a predefined search space, whereas the proposed ASSMEOA model innovatively modifies molecular structures by replacing fragments with those from an adaptive search space, thereby expanding the exploration ranges and potentially enhancing optimization outcomes. This differentiation may cause their limitations on optimizing specific properties of given molecules. All in all, the results indicate that molecules optimized by ASSMOEA have better properties. Although its SIM is not the largest, it is above the pre-set baseline.

### 3.5 Ablation experiments

ASSMEOA proposes two specific solutions for the search space. The first solution is the construction of molecule-specific search space. Instead of adopting a universal search space, a unique search space is designed for each molecule. This ensures that the fragments in the search space have the highest relevance to each lead molecule while controlling the size of the search space. The second step is the adaptive expansion of molecule-specific search space, it involves using an information fusion module to learn and discover molecules with better performance based on their own experience. These molecules are then added to the search space of the corresponding lead molecule, allowing for exploration of the unknown and desired space.

To determine the effectiveness of these steps in improving molecular optimization efficiency, we conducted an ablation experiment using the same two steps. The initial step involves removing the adaptive expansion of molecule-specific search space to fix the given high-quality fragments. This step is taken to determine whether there is a need to learn and generate superior molecules to expand the search space and improve the quality of the molecular fragment library. The second step is to remove the construction of molecule-specific search space and use generic preliminary molecules to construct the search space. This space is then used to verify whether adding similarity constraints to each molecule can narrow the search space and solve the problem of ineffective parts, which can affect search efficiency and help with the molecule optimization process.

In our experiments, we used the same optimization objectives and conditions as in the multi-property optimization experiments. We performed two experiments, QED+PLogP imp+SIM and QED+DRD2+SIM, to eliminate the effect of optimizing different properties due to the optimization.

**Figure 6.**
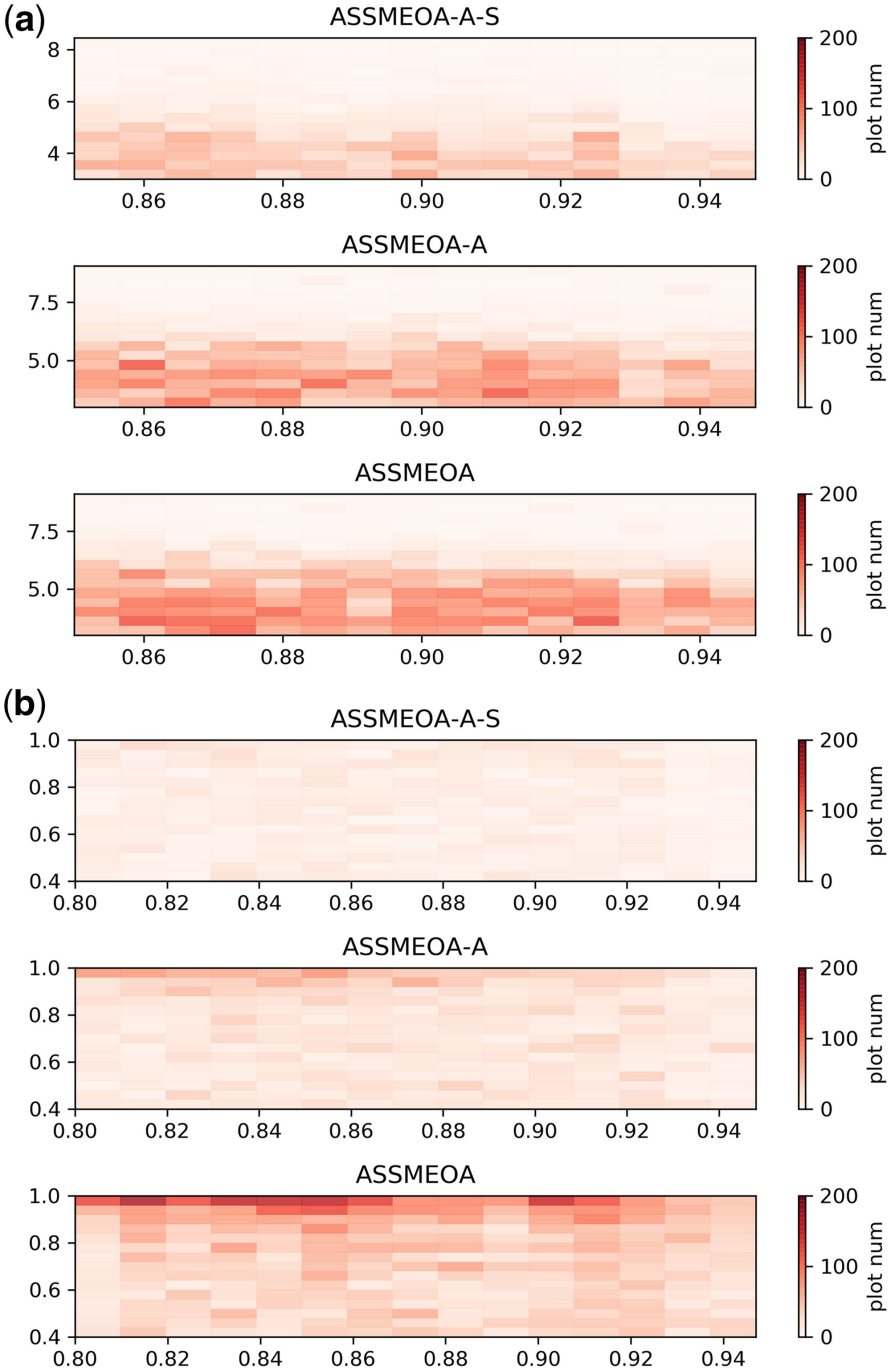
The results of ablation experiments in two tasks. (a) Task I; (b) Task II. ASSMEOA-A: the ablation of the adaptive expansion of molecule-specific search space; ASSMEOA-A-S: the ablation of the construction of molecule-specific search space.

The results of the two experiments are shown in Fig. 6. In each sub-figure, the deeper the plot is, the more successful molecules are optimized. The improvement from ASSMEOA-A-S to ASSMEOA-A and ASSMEOA is obvious. The heat maps of ASSMEOA-A-S are almost white in both Tasks I and II.

In Task I, the improvement from ASSMEOA-A-S to ASSMEOA-A is greater than that from ASSMEOA-A to ASSMEOA. The construction of molecule-specific search space plays an important role in the improvement. The majority reason is that optimizing QED and ${\text{PLogP}}_{\text{imp}}$ is easier in Task I, and the public molecule library contains more molecules with high values of QED and ${\text{PLogP}}_{\text{imp}}$. The construction of molecule-specific search space helps to focus on those molecules which are similar to lead molecule in optimization process, thus improves the optimization performance.

In Task II, the improvement from ASSMEOA-A to ASSMEOA is greater than that from ASSMEOA-A-S to ASSMEOA-A, that the adaptive expansion of molecule-specific search space plays a more important role. The majority reason is that optimizing DRD2 is harder than both QED and ${\text{PLogP}}_{\text{imp}}$, that the number of molecules with high value of DRD2 scores are very less in public molecule library. The adaptive expansion of molecule-specific search space helps to generate more novel molecules with high values of DRD2 scores and add them to the search space. The performance is improved finally.

### 3.6 Case study

**Figure 7.**
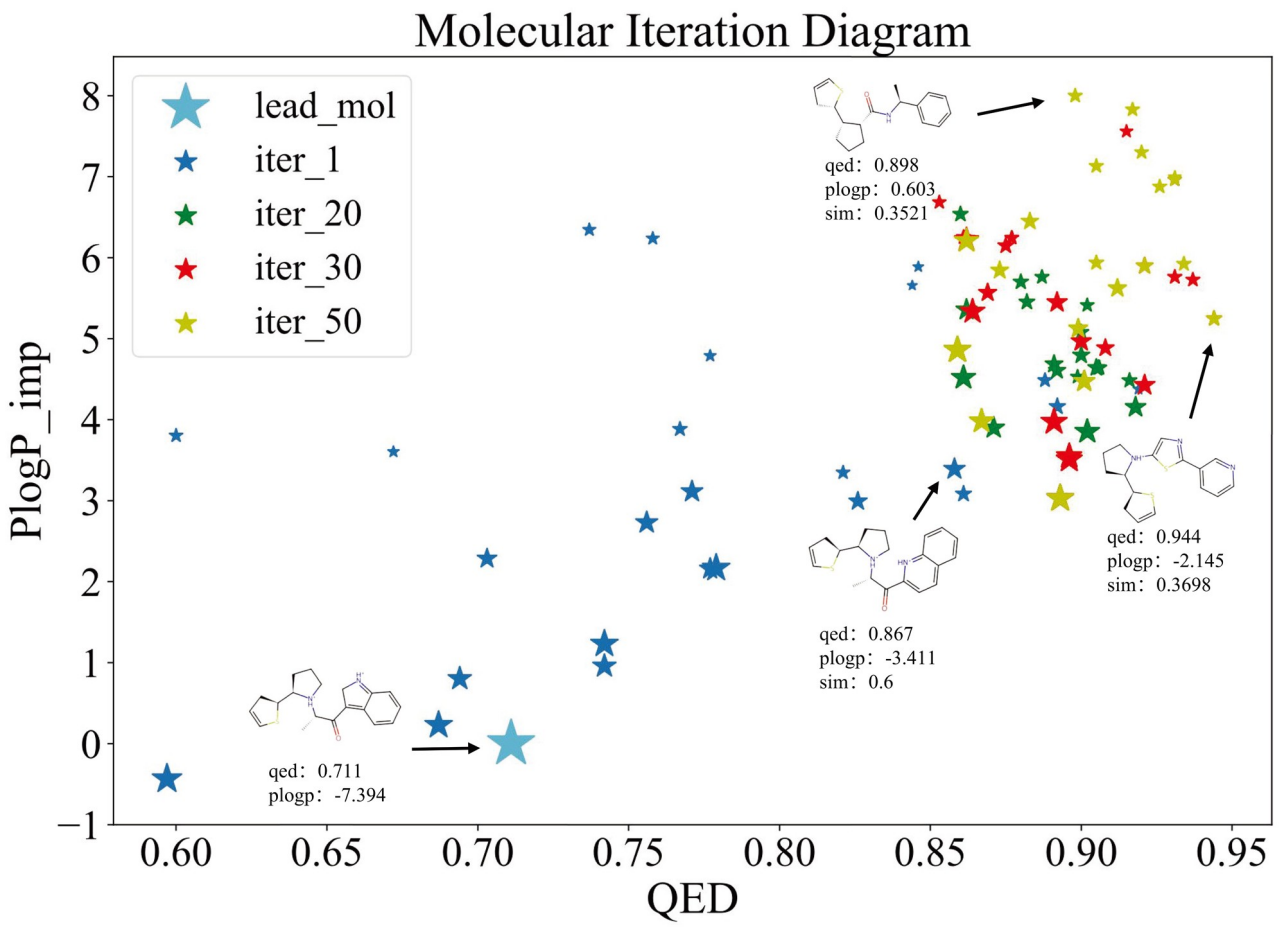
The visualization of optimization for a given molecule in the case study.

To illustrate the optimization process of ASSMEOA, we randomly chose a molecule $x_{0}$ from the ZINC dataset as a lead molecule, where the values of QED and PLogP of the molecule are, respectively, 0.711 and −7.394. The properties (i.e. QED, PLogP, and similarity) of the molecule during the iteration process are shown in Fig. 7. We can see that as the increase of iteration time, the values of QED and PLogP of the optimized molecule gradually improve.

**Figure 8.**
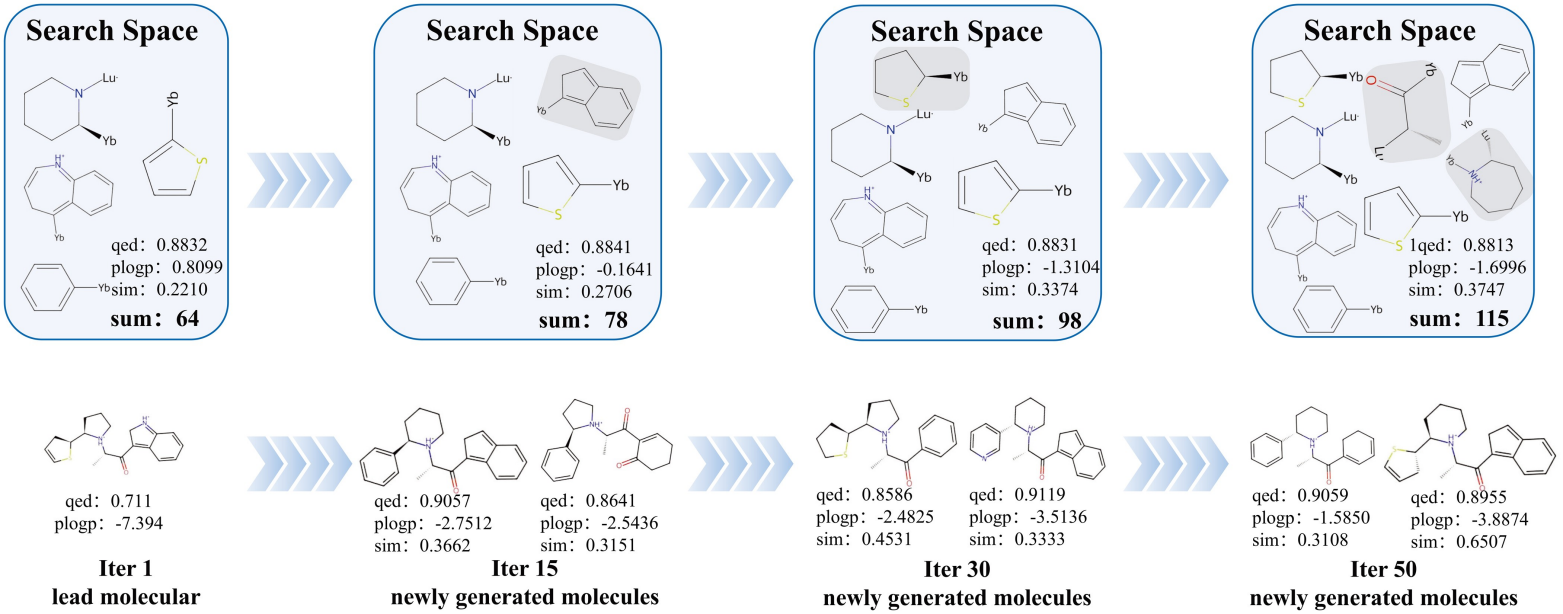
The search spaces and optimized molecules of four iterations during the optimization.

Moreover, we analyze the contribution of the adaptive molecule-specific fragment library of the above molecule. In this work, we use the average property of molecules to be the property of search space. As shown in Fig. 8, along the iterations such as the 15th, 30th, and 50th iteration, the similarity increases, and the QED values are stable. In the first iteration, the size of the search space is 64. In the end, it increases to 115. During the optimization, the molecule has more chance to attach with good fragments.

It is indicated that ASSMEOA effectively optimizes the lead molecule step by step. Meanwhile, the change in molecular properties gradually decreases in the late iteration, indicating that the molecular optimization has gradually reached convergence in the late molecular iteration.

In the view of the property of search space, our ASSMEOA achieves an improvement. In the original iteration, ASSMEOA selects 20 molecules which are most similar to the lead molecule $x_{0}$, construct a molecule-specific library of $x_{0}$, decompose the molecules into fragments to form a search space. The QED and PLogP of this search space are 0.8832 and 0.8099, respectively. However, its similarity to lead molecule is 0.2210, which indicates that SIM is hard to be improved based on the original search space. Benefiting from the Module (c) decomposes novel molecules with high SIM values into fragments and puts them into search space, molecule SIM increases during the optimization process. In Iter 15, 30, and 50, the SIM values are 0.2706, 0.3374, and 0.3747, respectively. Meanwhile, the values of QED and PLogP are stable.

In the view of the range of search space, our ASSMEOA increases the range and enhances the diversity. In the original iteration, ASSMEOA could only search 64 fragments in the search space. Although those 64 fragments are effective for optimization, some other high-quality fragments are missed. During the iteration, more novel fragments are added to the space. The search space is expanding to unknown and high-quality fragments. The number of fragments reach 115, finally. It increased by 79.7%, compared to the original search space. Molecules have more chances to explore fragments for optimization.

Therefore, ASSMEOA can easily explore molecules with better property and larger similarity to lead molecules, during the expansion and optimization of search space.

## 4 Conclusion

In this paper, we propose the ASSMEOA model to address some limitations in current combinatorial optimization models, such as the difficulty to explore the unknown search space, the decreased optimization efficiency resulting from useless fragments during the optimization, and the scarcity of desired substructures for certain properties. ASSMEOA takes into account the individual differences of each lead molecule and designs exclusive search spaces so that the fragments in the search space have the highest relevance to each lead molecule while controlling the size of the search space. Additionally, it employs an encoder–fusion–decoder structure in the optimization process to learn and unearth molecules with improved performance based on previous experience and incorporate them into the search space of the corresponding lead molecule to explore uncharted and more desirable territories. It further utilizes a tree structure to construct implicit connections within the fragment library and employs a dynamic mutation strategy to guide the mutations. The implicit connections, represented by trees, are employed to mitigate the random walk issue during the evolutionary process. Through multi-property optimization experiments, we demonstrate the effectiveness of ASSMEOA in improving the new energy of molecular optimization. The efficacy of the two proposed schemes for the search space is effectively demonstrated through ablation experiments.

## Data Availability

The code of the proposed ASSMEOA is freely available on the web at https://github.com/bbbbb-b/MEOAFST.
